# The Role of KRAS in Non-Small Cell Lung Cancer: From Molecular Background to Precision Medicine

**DOI:** 10.3390/ijms27188194

**Published:** 2026-09-15

**Authors:** Jadwiga Gaździcka, Karolina Gołąbek

**Affiliations:** Department of Medical and Molecular Biology, Faculty of Medical Sciences in Zabrze, Medical University of Silesia in Katowice, 19 Jordana St., 41-808 Zabrze, Poland; jgazdzicka@sum.edu.pl

**Keywords:** KRAS, non-small-cell lung cancer, NSCLC, mutations, polymorphisms, non-coding RNAs, KRAS inhibitors, targeted therapy

## Abstract

*KRAS* is one of the most frequently altered oncogenes in non-small cell lung cancer (NSCLC), playing a pivotal role in tumour initiation, progression, and therapeutic response. This review summarises the current understanding of the molecular mechanisms underlying KRAS-driven NSCLC, with particular emphasis on signalling pathways, genetic alterations, and their clinical implications. We discuss the spectrum of KRAS mutations and polymorphisms, highlighting their impact on disease biology, prognosis, and treatment outcomes. In addition, the review explores the contribution of epigenetic regulation to KRAS-mediated oncogenesis, focusing on the roles of non-coding RNAs, including microRNAs (miRNAs), long non-coding RNAs (lncRNAs), and circular RNAs (circRNAs), as well as post-translational modifications of the KRAS protein, such as methylation, phosphorylation, ubiquitination, nitrosylation, and acetylation. Recent advances in the development of KRAS-targeted therapies are presented, including mutation-specific and pan-KRAS inhibitors. Furthermore, we discuss combination treatment approaches designed to overcome intrinsic and acquired resistance by targeting complementary signalling pathways or modulating the tumour microenvironment. Collectively, this review highlights the complexity of KRAS biology in NSCLC and underscores the importance of integrating molecular, epigenetic, and therapeutic insights to advance precision medicine and improve clinical outcomes.

## 1. Introduction

Lung cancer remains one of the leading causes of cancer morbidity and mortality worldwide. Non-small cell lung cancer (NSCLC) accounts for approximately 85% of cases, with lung adenocarcinoma (LUAD) being the predominant histological subtype [1,2,3,4]. According to the 2021 WHO Classification of Thoracic Tumours, large cell carcinoma is now an uncommon diagnosis reserved for poorly differentiated non-small-cell carcinomas lacking glandular, squamous, or neuroendocrine differentiation [5]. Molecular characterisation has substantially changed NSCLC classification and treatment by enabling the identification of clinically relevant oncogenic drivers [6,7].

**Figure 1:**
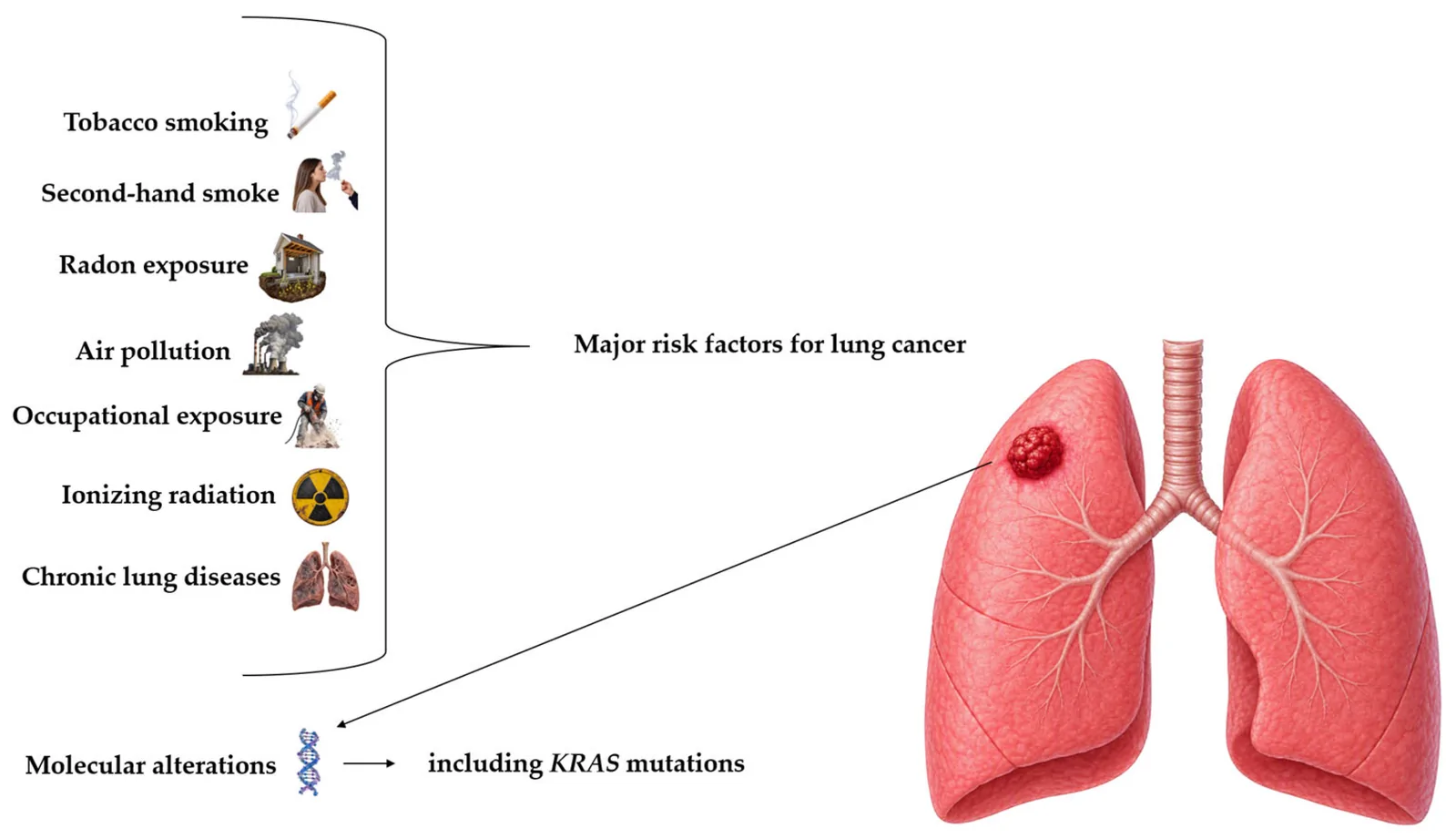
Major risk factors for lung cancer and molecular alterations, including KRAS mutations. Created by the authors in Microsoft Copilot (Microsoft Corporation; https://copilot.microsoft.com/; accessed on [7 August 2026]) and subsequently modified by the authors.

Lung cancer development is associated with multiple environmental, lifestyle, host-related risk factors, and genetic susceptibility [3,4]. These factors contribute to lung carcinogenesis and the accumulation of molecular alterations, including *KRAS* mutations (Figure 1).

*KRAS* (Kirsten Rat Sarcoma Viral Oncogene Homolog) is one of the most frequently mutated oncogenes in LUAD and plays a central role in tumour initiation, progression, metastatic behaviour, and therapeutic response [6,7]. The biological and clinical heterogeneity of KRAS-mutated NSCLC reflects not only the specific *KRAS* variant but also co-occurring genomic alterations and downstream signalling networks. In the Lung Cancer Mutation Consortium cohort, the median age at diagnosis of metastatic KRAS-mutant LUAD was 65 years, indicating that this molecular subgroup is not specifically enriched in very young patients [8].

Comprehensive molecular profiling is essential in advanced non-squamous NSCLC.

Targeted polymerase chain reaction (PCR)-based assays and next-generation sequencing (NGS) are used to detect clinically relevant alterations, whereas plasma circulating tumour DNA (ctDNA) provides a minimally invasive alternative when tissue is unavailable or insufficient; negative plasma results should, whenever feasible, be confirmed by tissue testing [9,10,11].

**Figure 2:**
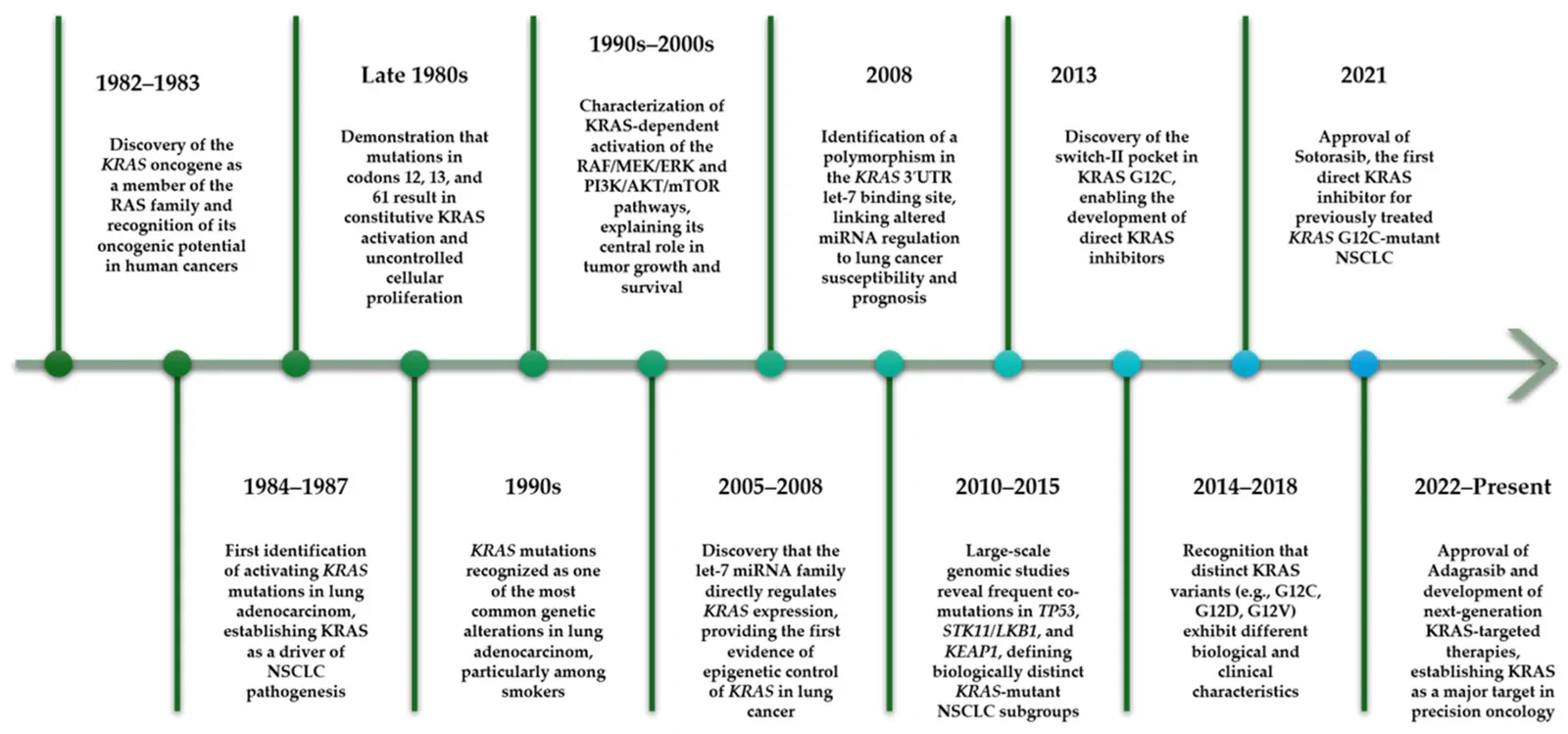
Key milestones regarding the KRAS oncogene and its role in the pathogenesis, diagnosis and treatment of NSCLC.

This review focuses on *KRAS* biology in NSCLC, including protein structure and signalling, somatic mutations and germline polymorphisms, epigenetic and post-translational regulation, and the evolving therapeutic landscape. Figure 2 highlights key milestones in the discovery and clinical relevance of *KRAS*.

## 2. Characterisation of the KRAS Gene and Protein

The *KRAS* locus is located on chromosome 12p12.1 [12]. Alternative processing of exon 4 generates two functional isoforms, KRAS4A and KRAS4B. Both isoforms are expressed in NSCLC, and differential KRAS4A/KRAS4B expression has been documented in advanced lung adenocarcinoma [13,14,15]. Experimental models further show that oncogenic KRAS4A can drive metastatic lung adenocarcinoma even in the absence of KRAS4B, supporting a biologically relevant role for KRAS4A in lung cancer [16]. Unlike KRAS4B, KRAS4A can undergo palmitoylation, a modification involved in membrane trafficking. The RAS palmitoylation/depalmitoylation cycle has therefore been proposed as a therapeutic vulnerability, although this strategy remains preclinical [17,18]. In addition, the human genome contains a non-functional pseudogene, KRAS1, located on chromosome 6 [15].

KRAS is a small guanosine triphosphatase (GTPase) with a molecular weight of approximately 21.6 kDa and comprising 188–189 amino acid residues, depending on the isoform. The protein consists of a highly structured N-terminal G-domain (residues 1–166) and a C-terminal hypervariable region (HVR). The G-domain adopts the characteristic GTPase fold, comprising six β-strands and five α-helices, and forms the nucleotide-binding and guanosine triphosphate (GTP) hydrolysis site essential for normal KRAS function. The C-terminal hypervariable region contains a hinge region and terminates with a conserved CAAX motif, which is required for post-translational modifications and membrane localisation [19]. The N-terminal G-domain of KRAS is responsible for guanine nucleotide binding and hydrolysis, thereby controlling the nucleotide-dependent activation state of the protein and its interactions with downstream effectors. Three functionally important regions within the G-domain include the phosphate-binding loop (P-loop; residues 10–14), Switch I (residues 30–40), and Switch II (residues 60–76). The P-loop interacts with the phosphate groups of GDP (Guanosine Diphosphate) and GTP, thereby stabilising nucleotide binding. In contrast, Switch I and Switch II undergo conformational changes during nucleotide exchange and GTP hydrolysis, enabling interactions with downstream effector proteins and regulatory factors. Together, these regions govern the activation state of KRAS and mediate signal transmission to downstream pathways. Mutations affecting the P-loop, Switch I, or Switch II frequently impair intrinsic GTPase activity or disrupt GTP hydrolysis, leading to prolonged KRAS activation and enhanced oncogenic signalling [18]. The structural flexibility of the G-domain enables KRAS to interact with a diverse range of effector and regulatory proteins involved in cellular signalling. Effector recognition is primarily mediated by GTP-dependent conformational changes within Switch I and Switch II, which form the binding interface for proteins such as rapidly accelerated fibrosarcoma kinases (RAF kinases) and phosphoinositide 3-kinase (PI3K). In contrast, guanine nucleotide exchange factors (GEFs), including SOS1, promote the release of GDP and facilitate GTP loading, thereby activating KRAS. GTPase-activating proteins (GAPs), such as neurofibromin 1 (NF1), accelerate GTP hydrolysis and return KRAS to its inactive GDP-bound state. These interactions depend on specific structural features within the G-domain that ensure the spatial and temporal regulation of signal transduction in response to extracellular stimuli [19].

HVR forms the C-terminal portion of KRAS and plays a central role in determining its intracellular localisation and membrane association. The HVR terminates with a conserved CAAX motif that undergoes farnesylation, an essential post-translational modification required for membrane targeting and biological activity [20]. Additional modifications contribute to the distinct membrane-targeting properties of the KRAS isoforms. KRAS4A can undergo palmitoylation, whereas KRAS4B contains a lysine-rich polybasic region that promotes electrostatic interactions with negatively charged membrane phospholipids. These isoform-specific features influence membrane trafficking, subcellular distribution, and interactions with signalling complexes, ultimately contributing to differences in downstream signal transduction [18].

## 3. KRAS Signalling Pathways and Molecular Functions

KRAS functions as a molecular switch that cycles between an inactive GDP-bound state and an active GTP-bound state. The transition between these conformational states regulates the transmission of intracellular signals from activated cell-surface receptors to downstream effector pathways [21,22]. KRAS activation is initiated following the binding of extracellular ligands, including growth factors, cytokines, and hormones, to cell-surface receptors, most commonly receptor tyrosine kinases (RTKs) such as the epidermal growth factor receptor (EGFR). Ligand binding induces receptor dimerisation and autophosphorylation of specific tyrosine residues within the intracellular domain. These phosphorylated residues serve as docking sites for adaptor proteins, including growth factor receptor-bound protein 2 (GRB2), which recruits GEFs, most notably SOS1, to the plasma membrane. SOS1 promotes the release of GDP from KRAS, allowing GTP binding due to the higher intracellular concentration of GTP relative to GDP. GTP loading induces conformational rearrangements within the Switch I and Switch II regions, generating the active form of KRAS capable of interacting with downstream effector proteins. Under physiological conditions, KRAS possesses intrinsic GTPase activity and can hydrolyse bound GTP to GDP. However, this process is relatively inefficient and is markedly accelerated by GAPs, such as NF1, which facilitate signal termination [21,22,23].

Once activated, GTP-bound KRAS interacts with multiple downstream effectors and regulates several signalling pathways, including the RAF/MEK/ERK (MAPK) pathway, the PI3K/AKT/mTOR pathway, the RAL/GDS pathway, and the phospholipase C epsilon (PLCε) pathway [24,25,26,27,28].

In the RAF/MEK/ERK pathway, activated KRAS recruits RAF kinases, including BRAF, to the plasma membrane, promoting their activation. RAF subsequently phosphorylates and activates MEK1 and MEK2, which in turn phosphorylate ERK1 and ERK2. Activated ERK translocates to the nucleus, where it regulates transcription factors such as MYC (MYC Proto-Oncogene) and ELK1 (ETS Transcription Factor ELK1), thereby controlling the expression of genes involved in cell proliferation, differentiation, and survival [25,26].

The PI3K/AKT/mTOR pathway plays a central role in cellular growth, metabolism, and survival. In this pathway, KRAS directly interacts with and activates PI3K, resulting in the generation of phosphatidylinositol-3,4,5-trisphosphate (PIP3). PIP3 recruits and activates protein kinase B (AKT), which promotes cell survival through the inhibition of apoptotic signalling and stimulates the mammalian target of rapamycin (mTOR), a key regulator of protein synthesis and cellular growth [27].

KRAS also activates the RAL/GDS pathway through interaction with RAL guanine nucleotide dissociation stimulator (RALGDS). This signalling axis regulates the activity of the small GTPases RalA and RalB and contributes to cytoskeletal remodelling, vesicle trafficking, cell migration, and metastatic progression [24]. In KRAS-driven human cancer cells, RalA-dependent phospholipase D (PLD) activity can provide a pro-survival signal; this has also been demonstrated in a KRAS-mutant lung cancer cell model [29].

In addition, KRAS can directly interact with PLCε through its Ras-association (RA) domains. Activated PLCε hydrolyses phosphatidylinositol-4,5-bisphosphate (PIP2), generating the second messengers diacylglycerol (DAG) and inositol-1,4,5-trisphosphate (IP3). These molecules promote calcium signalling and activation of protein kinase C (PKC) family members, thereby contributing to cellular proliferation and survival [28].

A further branch relevant to tumour invasion involves RAC1. RAS can engage the guanine-nucleotide exchange factor TIAM1, which activates RAC1 and promotes actin-cytoskeleton reorganisation, motility, and invasive behaviour. In lung cancer, TIAM1 accumulation is also linked to growth-factor signalling, supporting a potential role in adaptive therapeutic responses [30,31].

KRAS effector pathways do not function independently. Reciprocal feedback and crosstalk between RAF/MEK/ERK and PI3K/AKT/mTOR signalling can maintain proliferative and survival signals when one branch is pharmacologically inhibited. This network plasticity contributes to intrinsic and acquired resistance and provides a rationale for effective combination strategies [25,26,27].

KRAS-driven signalling can additionally engage TBK1-dependent NF-κB activity, which supports survival of KRAS-dependent cancer cells [32]. Basal autophagy is also a relevant stress-adaptation mechanism in KRAS-driven NSCLC and can intersect with non-canonical NF-κB signalling [33]. ULK1, an initiating kinase of autophagy, has therefore been investigated as a therapeutic vulnerability; pharmacological ULK1 inhibition can sensitise KRAS-mutant lung cancer cells to nutrient stress [34].

Metabolic rewiring is closely integrated with KRAS signalling. KRAS-mutant NSCLC can increase glycolytic and glutamine-dependent metabolism, while mTOR coordinates nutrient sensing and anabolic growth. KRAS G12D-driven NSCLC shows a particularly strong dependency on PI3K/AKT/mTOR signalling, supporting mutation-specific combinations of direct KRAS inhibition with pathway-directed approaches [15,35].

**Figure 3:**
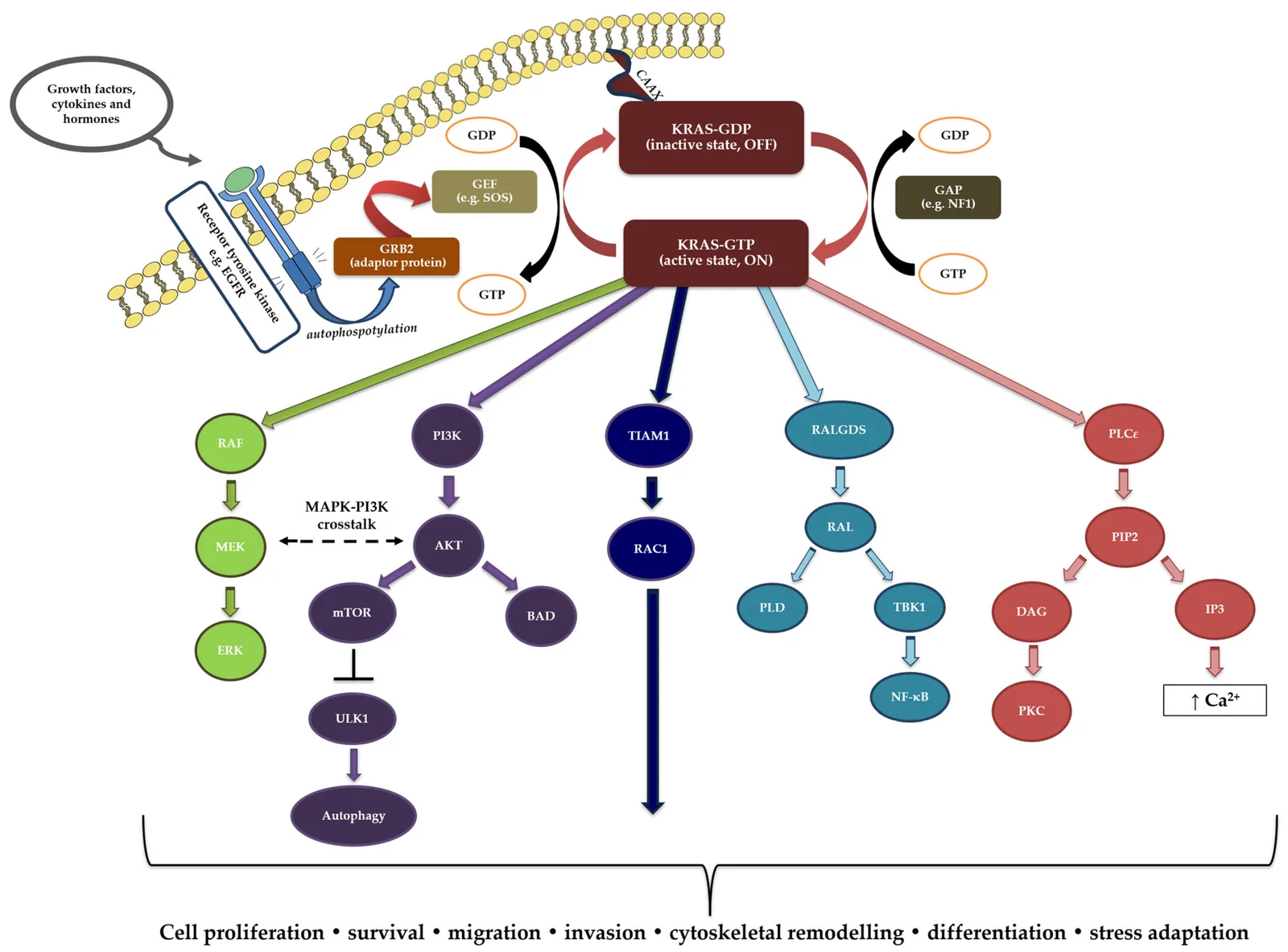
The mechanism of KRAS activation and its principal downstream signalling pathways. (Abbreviations: BAD: BCL2-Associated Agonist of Cell Death; ERK: Extracellular Signal-Regulated Kinase; MEK: Mitogen-Activated Protein Kinase Kinase (MAPKK); NF-κB: Nuclear Factor Kappa B; RAL: Ras-Like GTPase.

The mechanism of KRAS activation and its principal downstream signalling pathways are summarised in Figure 3.

## 4. KRAS Gene Alterations and Their Role in NSCLC Pathogenesis

### 4.1. Mutations

**Figure 4:**
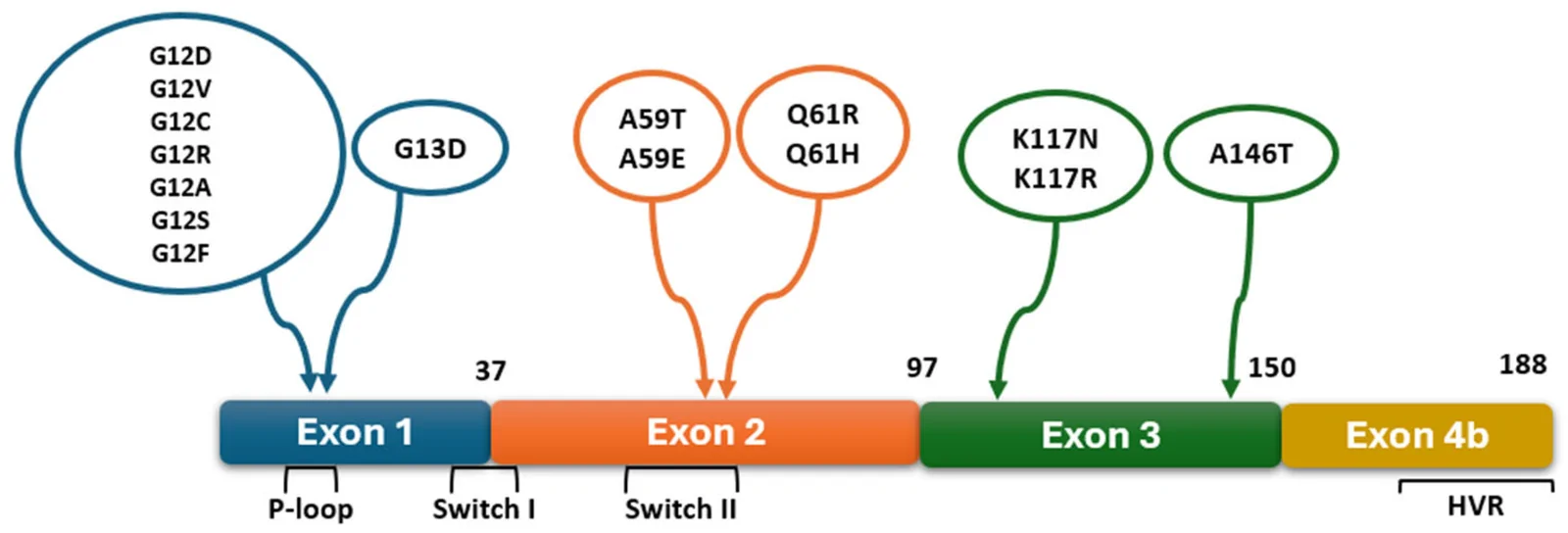
Distribution of the most common *KRAS* mutation sites across the protein sequence and their relationships to exons and functional regions.

**Figure 5:**
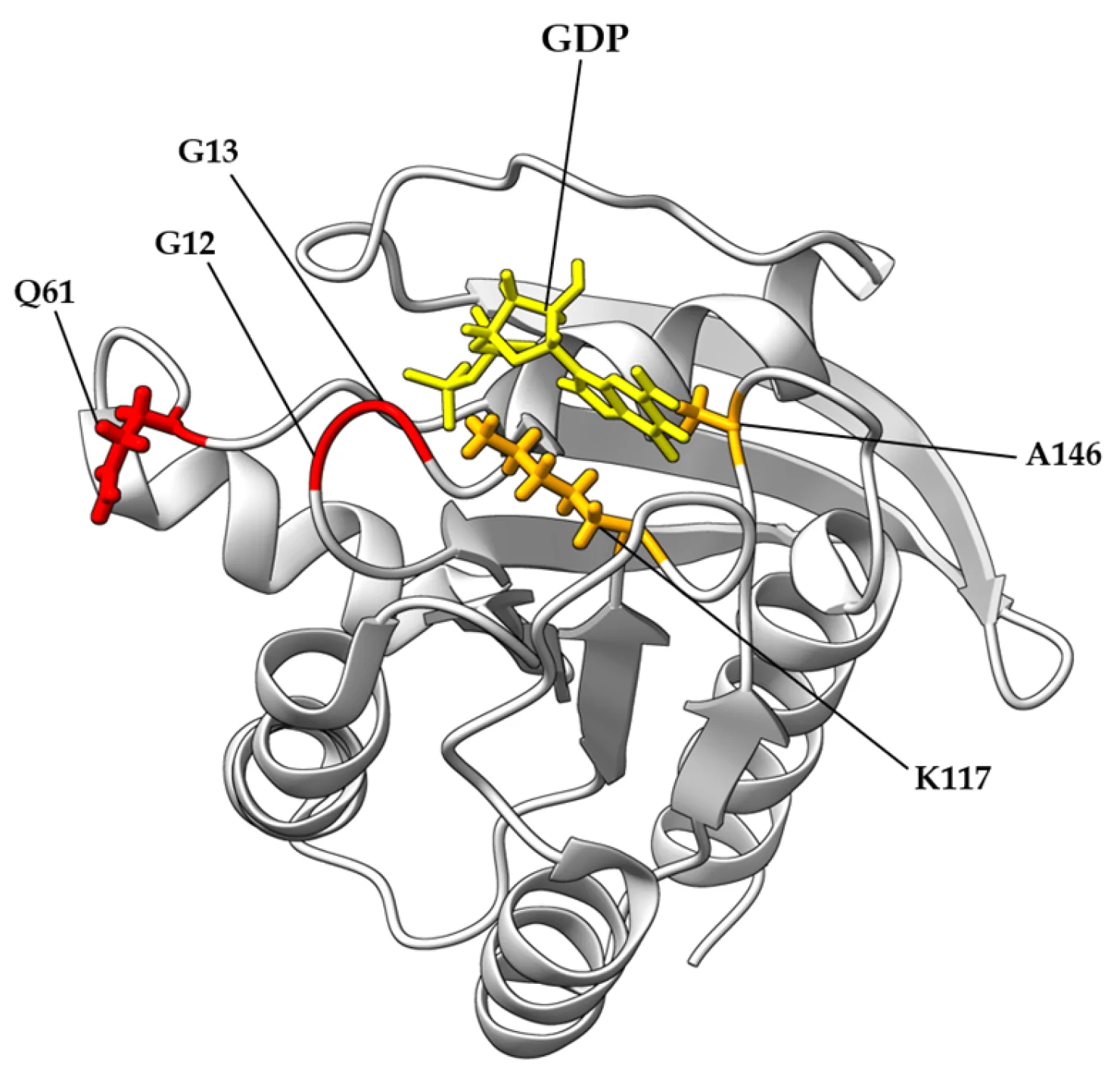
Structural localisation of KRAS mutation hotspots. Three-dimensional structure of wild-type KRAS bound to GDP (PDB ID: 6MBT). Major mutational hotspots G12, G13, and Q61 are highlighted in red, whereas the less frequent hotspot residues K117 and A146 are shown in orange. GDP is shown in yellow. Created by the authors using UCSF ChimeraX (version 1.12).

*KRAS* mutations represent one of the most frequent oncogenic alterations in NSCLC, particularly in lung adenocarcinoma [36,37]. In contrast, KRAS mutations are uncommon in lung squamous cell carcinoma (LUSC), with reported frequencies of approximately 4–4.4% [38,39]. In NSCLC, particularly in adenocarcinoma, prevalence is estimated at approximately 25–30% in Western populations, whereas lower frequencies, ranging from 15% to 25%, have been reported in Asian populations [36,37]. Approximately 90% of *KRAS* mutations occur in codon 12, with less frequent alterations affecting codons 13 and 61 [36,40]. Among these, the G12C substitution is the predominant variant, accounting for approximately 40–45% of all *KRAS* mutations, followed by G12V (approximately 20–25%) and G12D (approximately 15–20%) [36,37,41]. Less frequent activating KRAS mutations may also involve codons 117 and 146, which, together with codons 12, 13, and 61, represent recurrently mutated residues of KRAS [42,43]. The distribution of the most common KRAS mutation sites across the protein sequence and their relationship to individual exons and functional regions are illustrated in Figure 4. To complement this linear representation, Figure 5 shows the spatial localisation of the major mutational hotspots G12, G13, and Q61, together with the less frequent hotspots K117 and A146, within the three-dimensional structure of GDP-bound KRAS (Protein Data Bank (PDB) ID: 6MBT), visualised using UCSF ChimeraX (version 1.12) [44].

The occurrence of *KRAS* mutations is strongly associated with tobacco exposure. More than 90% of patients harbouring *KRAS*-mutated tumours are current or former smokers [37,45]. The G12C variant is particularly enriched in individuals with a history of heavy tobacco use, whereas G12D mutations are more frequently observed in never-smokers [36,37]. KRAS-mutated NSCLC also frequently exhibits relatively high tumour mutational burden (TMB), particularly in smoking-associated disease, a biological feature with potential relevance to immunotherapy response [41,46].

Several studies have also suggested sex-related differences in mutation frequency, with some reports indicating a slightly higher prevalence among women; however, these observations have not been consistently confirmed across clinical cohorts [36,47].

In the Lung Cancer Mutation Consortium, the median age of patients with metastatic KRAS-mutant lung adenocarcinoma was 65 years [8], reinforcing that KRAS-mutated NSCLC is typically diagnosed in older adults rather than being specifically enriched in very young patients.

Oncogenic KRAS mutations, particularly substitutions affecting codon 12, impair intrinsic and/or GAP-stimulated GTP hydrolysis and increase the fraction of GTP-bound KRAS [37,48]. Consequently, mutant KRAS continuously stimulates downstream signalling pathways, most notably RAF/MEK/ERK and PI3K/AKT/mTOR, even in the absence of external growth signals [41,49].

Persistent activation of these pathways promotes uncontrolled cellular proliferation, enhances survival signalling, and suppresses apoptotic processes. In addition, *KRAS*-driven tumours undergo metabolic reprogramming characterised by increased glycolysis and glutamine utilisation, which support the energetic and biosynthetic demands of rapidly proliferating cancer cells. Experimental studies using genetically engineered mouse models have demonstrated that mutant *KRAS* can act as an early driver of lung tumourigenesis. Notably, *KRAS* G12D has been shown to induce tumour formation more efficiently than *KRAS* G12C in several preclinical models [35,37,49,50,51].

The greater tumour-initiating potency reported for KRAS G12D compared with KRAS G12C has been associated with stronger PI3K-AKT-mTOR activation during tumour initiation. In established models, G12D tumours also retain a greater dependency on this axis, helping to explain mutation-specific vulnerabilities and supporting combinations of emerging G12D inhibitors with the AKT/mTOR-pathway blockade [35].

Beyond its direct effects on tumour cell proliferation, mutant *KRAS* contributes to epithelial–mesenchymal transition (EMT), remodelling of the tumour microenvironment, and immune evasion. These processes facilitate tumour invasion, metastatic dissemination, and resistance to anti-tumour immune responses [50,52].

Although the prognostic significance of *KRAS* mutations remains a matter of debate, the majority of studies indicate that *KRAS*-mutated NSCLC is associated with less favourable clinical outcomes compared with *KRAS* wild-type disease. In particular, *KRAS* G12D has been linked to shorter overall survival (OS) and progression-free survival (PFS). In patients with early-stage NSCLC (stages I–II), *KRAS* mutations, especially when accompanied by *TP53* alterations, are associated with an increased risk of disease recurrence following curative treatment [37,41,53,54].

*KRAS* mutations also influence metastatic patterns and organ-specific dissemination. In contrast to tumours harbouring *EGFR* mutations or *ALK* rearrangements, which frequently metastasise to the central nervous system, *KRAS*-mutated tumours exhibit a greater propensity for metastasis to the bone, liver, adrenal glands, and lungs [55]. Proposed mechanisms underlying this organotropism include EMT activation and exosome-mediated formation of pre-metastatic niches [36,55]. Furthermore, the *KRAS* G12V variant has been associated with an increased risk of early disease recurrence [37].

The biological and clinical behaviour of *KRAS*-mutated tumours is additionally shaped by co-occurring genomic alterations. Therefore, assessment of *KRAS* mutation status alone is often insufficient to fully characterise tumour biology. Co-mutations are detected in approximately one-third of *KRAS*-mutated NSCLC cases and may substantially influence prognosis, tumour microenvironment composition, therapeutic response, and disease progression. The most frequently reported co-mutations involve *TP53* (Tumour Protein p53), *STK11* (Serine/Threonine Kinase 11) also known as *LKB1* (Liver Kinase B1), *KEAP1* (Kelch-Like ECH-Associated Protein 1), *SMARCA4* (SWI/SNF-Related Matrix-Associated Actin-Dependent Regulator of Chromatin Subfamily A Member 4), and *CDKN2A/B* (Cyclin-Dependent Kinase Inhibitor 2A and 2B) [45]. The clinical and biological characteristics associated with these co-mutations are summarised in Table 1.

KRAS is classically mutually exclusive with other dominant oncogenic drivers, particularly sensitising EGFR mutations, ALK rearrangements, and ROS1 fusions. For EGFR and KRAS in particular, this mutual exclusivity may reflect functional redundancy of downstream signalling and negative selection against simultaneous strong activation of the EGFR/RAS/MAPK axis. Experimental studies have also suggested that concurrent oncogenic EGFR and KRAS signalling may induce a synthetic-lethal state, providing a biological explanation for the rarity of true double-driver tumours. Nevertheless, modern NGS studies have demonstrated that true co-occurrence can occur rarely, potentially reflecting biological heterogeneity or subclonal evolution. Such cases remain important for molecular classification and treatment selection and should be interpreted in light of variant pathogenicity, allele fraction, and assay quality rather than assumed to be impossible [59,60,61,62,63,64].

### 4.2. Germline Polymorphisms and Other KRAS Sequence Variants

For clarity, the term polymorphism is used here for inherited germline variants that are present at appreciable frequency in a population, whereas tumour-acquired substitutions are described as somatic variants or mutations. This distinction is particularly important in tumour-only NGS, where the germline or somatic origin of a detected variant cannot always be established without analysis of matched normal DNA. Variants of uncertain significance should not be classified as pathogenic solely on the basis of in silico prediction [62,63].

Single-nucleotide variants (SNVs) can be synonymous or nonsynonymous. In germline genetics, the term single-nucleotide polymorphism (SNP) is generally reserved for variants that are relatively common in a population, whereas tumour sequencing reports commonly use the broader term variant or SNV irrespective of population frequency. Nonsynonymous variants may affect protein structure, stability, and function; however, computational predictions alone are insufficient to establish pathogenicity [62,65].

In silico analysis of KRAS single-nucleotide variants using five bioinformatics tools demonstrated that four variants (rs868857258, rs1463850736, rs754870563, and rs138669124) were consistently predicted to be deleterious and damaging by all applied tools. Furthermore, protein stability analysis indicated that all four variants may decrease the stability of the KRAS protein. Structural and functional analyses suggested that these substitutions could affect KRAS conformation and function, potentially influencing processes such as GTP hydrolysis and effector interactions. Consequently, these variants were predicted to have potential relevance to KRAS-driven cancers. However, these findings are based solely on computational predictions and, therefore, require experimental validation to confirm their biological and clinical significance [66]. Another study employed several bioinformatics tools to screen nonsynonymous *KRAS* variants and identified ten variants (rs387907206, rs121913527, rs387907205, rs104894359, rs104894364, rs104894366, rs730880472, rs121913538, rs776785730, and rs1555195579) as highly pathogenic and/or deleterious. Structural analyses suggested that these variants may impair KRAS protein structure and stability, potentially affecting its function [67].

*KRAS* sequence variants have also been investigated in the context of lung cancer using computational approaches. Variants rs121913236 (Q22K) and rs121913240 (Q61P and Q61R) were identified as highly deleterious and associated with lung tumours. These variants were suggested to alter the structural stability and flexibility of KRAS compared with the wild-type protein, potentially affecting its biological function [68].

Studies investigating KRAS sequence variants in NSCLC remain limited, and tumour-derived hotspot substitutions should not be classified as germline polymorphisms unless matched normal material establishes germline origin. Dhakar et al. analysed the *KRAS* variant rs121913240 (Q61L) using datasets from LUAD and LUSC patients. Their results classified rs121913240 as a pathogenic/deleterious variant and suggested that this substitution increases KRAS protein stability [69]. On the other hand, Song et al. analysed *KRAS* variants in patients with non-squamous NSCLC with and without brain metastases. They found that rs121913529 and rs121913240 occurred more frequently in patients with brain metastases. Moreover, patients harbouring rs121913240 had a poorer prognosis than those harbouring rs121913529. The co-occurrence of these two variants in patients with brain metastases treated with bevacizumab-based combination therapy was associated with shorter overall survival and poorer clinical outcomes [70]. rs121913529 was also identified in NSCLC patients using whole-exome sequencing datasets. The two rs121913529 variants, G12V and G12D, were predicted to be pathogenic and to decrease KRAS protein stability [71]. In addition, the *KRAS* variant rs1137282 was associated with relapse-free survival (RFS) in NSCLC patients who underwent surgical resection. Patients carrying the AA genotype showed a higher 3-year RFS rate than those with AG or GG genotypes. These findings suggest that rs1137282 may serve as a prognostic marker of recurrence in resectable NSCLC [72].

SNPs located within let-7 complementary sites (LCSs) in the 3′-untranslated region (3′UTR) of *KRAS* may influence cancer risk by affecting microRNA binding and consequently altering *KRAS* expression. The *KRAS* 3′UTR contains the rs61764370 polymorphism, which involves a T to G substitution within the LCS6 region [73]. This polymorphism was first investigated by Chin et al., who reported that individuals carrying the TG/GG genotypes had an increased risk of NSCLC among moderate smokers. The study also demonstrated that the variant was associated with increased *KRAS* expression in vitro [74]. However, no significant association between rs61764370 and survival outcomes in NSCLC patients was observed in a subsequent study [75]. Likewise, a later meta-analysis found no association between rs61764370 and NSCLC risk in Caucasian populations [73]. In contrast, a study conducted in an Iranian population found that the GT genotype and the G allele of rs61764370 were significantly associated with an increased risk of lung cancer [76]. Additionally, rs712 is another polymorphism located in the 3′UTR of the *KRAS* gene. The GG genotype of rs712 was associated with a significantly increased risk of lung cancer among Chinese patients with chronic obstructive pulmonary disease (COPD) compared with the TT and TG genotypes [77]. In an Iranian population, however, the GT and TT genotypes of rs712 were associated with an increased risk of lung cancer, while the TT genotype was additionally associated with the presence of metastasis [78].

In summary, the available literature includes a mixture of confirmed germline polymorphisms, tumour-detected somatic variants, and variants analysed only computationally. These variants, together with their reported origin, study context, and major limitations, are summarised in Table 2. Therefore, their evidentiary strength is heterogeneous. Allele frequencies and linkage patterns also differ among populations, limiting direct generalisation of associations from single ethnic or geographic cohorts. Future studies should distinguish germline from somatic origin using matched normal material when possible and validate computational predictions experimentally and clinically [62,63].

## 5. Epigenetic Regulation of KRAS in NSCLC

Epigenetic regulation comprises reversible molecular processes that alter gene expression or protein function without changing the underlying DNA sequence. In KRAS-driven NSCLC, relevant mechanisms include DNA methylation and chromatin regulation, non-coding RNA-mediated control of KRAS transcripts, and post-translational modifications that alter KRAS stability, localisation, and signalling. Because these processes can be reversible, they may provide biomarkers as well as therapeutic vulnerabilities [18,79,80].

### 5.1. Non-Coding RNA

Non-coding RNAs (ncRNAs) are RNA molecules that do not encode proteins but play important regulatory roles in numerous cellular processes, including gene expression, RNA stability and degradation. Based on their size and structure, ncRNAs can be classified into several groups, including long non-coding RNAs (lncRNAs) and circular RNAs (circRNAs), which are typically longer than 200 nucleotides, as well as shorter ncRNAs (18–200 nucleotides), such as microRNAs (miRNAs), small interfering RNAs (siRNAs) and PIWI-interacting RNAs (piRNAs) [80,81]. Dysregulation of ncRNAs has been implicated in the pathogenesis of numerous diseases, including lung cancer, in which lncRNAs, miRNAs and circRNAs have been extensively studied [81,82]. This section focuses on ncRNAs that directly interact with *KRAS* and contribute to lung carcinogenesis.

#### 5.1.1. MiRNA

MiRNAs are one of the subgroups of non-coding RNAs. They are typically 18–25 nucleotides in length. miRNAs most frequently regulate gene expression by binding to complementary sequences within the 3′UTR of target mRNAs, leading to translational repression and/or mRNA degradation. Although the majority of miRNA–mRNA interactions occur within the 3′UTR, miRNAs may also bind to the 5′UTR or coding regions of mRNAs, as well as interact with promoter regions under specific conditions [83]. Therefore, miRNAs may function either as oncogenic miRNAs or tumour-suppressive miRNAs and influence numerous cellular processes, including proliferation, growth, apoptosis, and differentiation [84]. Among the miRNAs reported to interact with the *KRAS* 3′UTR in NSCLC are members of the let-7 family [85], miR-1205 [86], miR-193a-3p [87,88], miR-199b [89], miR-181a-5p [90], miR-202 [91] and miR-101-3p [92], all of which have been implicated in post-transcriptional regulation of *KRAS* expression.

Let-7 is one of the microRNAs that regulates the RAS family by directly targeting *KRAS* [85]. Decreased expression of let-7 has been widely reported in lung cancer, and its downregulation has been associated with increased *KRAS* expression [85,93], shorter survival in patients with NSCLC [93,94,95], whereas overexpression of let-7 has been reported to inhibit tumour progression [96]. Similarly, let-7g, a member of the let-7 family, reduced cell proliferation both in vitro (A549 cells) and in vivo [97]. Furthermore, decreased let-7g expression in NSCLC patients was associated with the presence of lymph node metastases [98]. Prolonged cigarette smoke exposure was shown to decrease let-7a expression and increase the frequency of G/T transversions in codons 12 and 13 of *KRAS* in cigarette smoke-exposed mice. Moreover, the study revealed a negative correlation between let-7a expression and *KRAS* mutations in tumour-bearing mice, supporting the involvement of multiple molecular mechanisms, including both genetic and epigenetic alterations, in lung carcinogenesis [99].

MiR-1205 was found to directly target *KRAS* mRNA and suppress *KRAS* expression. In NSCLC patient samples, miR-1205 expression was significantly reduced and inversely correlated with *KRAS* expression. It was demonstrated that miR-1205 decreased *KRAS* mRNA and protein levels in A549 and H1975 cells. These findings suggest that miR-1205 acts as a tumour suppressor and may have therapeutic potential in NSCLC. In vivo, restoration of miR-1205 reduced tumour growth and decreased the protein expressions of KRAS, MDM4 (Mouse Double Minute 4, Human Homolog Of; P53-Binding Protein) and E2F1 (E2F Transcription Factor 1). Therefore, hsa-miR-1205 may represent a potential therapeutic target in NSCLC [86].

Another microRNA that directly regulates *KRAS* mRNA is miR-193a-3p. It was demonstrated that miR-193a-3p expression was decreased in NSCLC tissues, whereas its restoration in vitro (A549 and H1975 cells) and in vivo reduced cell proliferation and migration. The study further showed that miR-193a-3p directly targets *KRAS* and reduces KRAS protein expression at the post-transcriptional level without significantly affecting *KRAS* mRNA levels [87]. Two functionally independent binding sites for miR-193a-3p were identified within the 3′UTR of *KRAS*. Moreover, an inverse correlation between miR-193a-3p expression and *KRAS* expression was observed in A549 cells. Functional analyses demonstrated that miR-193a-3p inhibited proliferation and cell-cycle progression through direct targeting of *KRAS*, suggesting its potential utility as a therapeutic strategy for KRAS-driven lung cancer [88]. Using an in silico target prediction approach, hsa-miR-193a-3p and hsa-miR-193b-3p were identified among the candidate miRNAs predicted to bind *KRAS* mRNA in smoking-associated lung cancer [100].

Additionally, miR-199b directly suppresses *KRAS* mRNA and protein expression. An inverse correlation between mutant *KRAS* and miR-199b expression was demonstrated in NSCLC specimens and cell lines. Furthermore, ectopic expression of *KRAS*(G12D) reduced miR-199b levels, whereas *KRAS* silencing increased miR-199b expression. Mechanistically, mutant *KRAS* enhanced miR-199b promoter methylation, leading to suppression of miR-199b expression and promoting NSCLC growth and metastasis. The study also found that miR-199b acts as a tumour suppressor by directly targeting *KRAS*, *KSR2* (Kinase Suppressor Of Ras 2), *PIK3R1* (Phosphoinositide-3-Kinase Regulatory Subunit 1), *AKT1* and *RHEB1* (RHEB Pseudogene 1), thereby inhibiting PI3K/AKT and ERK signalling. These findings suggest that restoration of miR-199b may represent a potential therapeutic strategy for *KRAS*-mutated NSCLC [89].

MiR-181a-5p has been proposed as a tumour-suppressive miRNA in NSCLC. It was demonstrated that miR-181a-5p directly regulates *KRAS*, thereby reducing *KRAS* expression. Reduced miR-181a-5p expression was observed in murine NSCLC models, human NSCLC tissues and several NSCLC cell lines, particularly A549, H23 and H1299. Restoration of miR-181a-5p expression inhibited proliferation and migration of A549 cells. Based on these findings, miR-181a-5p was proposed as a potential therapeutic target in NSCLC [90].

It was demonstrated that miR-202 directly targets *KRAS* in NSCLC. Bioinformatic analyses identified a conserved miR-202 binding site within the 3′UTR of *KRAS* mRNA, which was subsequently confirmed by luciferase reporter assays. Overexpression of miR-202 reduced KRAS protein expression in *KRAS*-mutant NSCLC cell lines (NCI-H441 and A549). It was found that miR-202 expression was significantly decreased in NSCLC tumour tissues and in *KRAS*-mutant NSCLC cells compared with non-tumour controls. This study showed that restoration of miR-202 enhanced cisplatin-induced apoptosis by suppressing the KRAS/MAPK signalling pathway. The authors suggested that combining miR-202 restoration with platinum-based chemotherapy may represent a potential therapeutic strategy for *KRAS*-mutant NSCLC [91].

In NSCLC, *KRAS* mRNA was identified as a direct target of miR-101-3p. Overexpression of this microRNA reduced expression of KRAS protein in NSCLC. MiR-101-3p was shown to be downregulated by circular RNA mediator of cell motility 1 (circ-MEMO1), resulting in increased KRAS expression, which contributed to NSCLC progression. This study revealed that the circ-MEMO1/miR-101-3p/KRAS axis plays an important role in NSCLC development and may represent a potential therapeutic target [92].

In the UTR region of the *KRAS* gene, changes in single nucleotides may occur, which affects microRNA binding. The 3′UTR region of the *KRAS* gene was analysed in 39 NSCLC patients using sequencing and the bioinformatic tool miRDB, resulting in the identification of a total of 53 variants within *KRAS* 3′UTR. Some of them impacted the number of miRNAs targeting this UTR; nine variants reduced the amount of miRNAs, one variant increased the predicted number of miRNAs binding to the *KRAS* 3′UTR, while 43 had no impact on the number of miRNAs targeting this 3′UTR. Additionally, nine detected variants of 3′UTR *KRAS* blocked binding of a total of 13 miRNAs (hsa-miR-3908, hsa-miR-6514-3p, hsa-miR-124-3p, hsa-miR-3714, hsa-miR-3910, hsa-miR-506-3p, hsa-miR-4283, hsa-miR-4489, hsa-miR-3059-5p, hsa-miR-4426, hsa-miR-4647, hsa-miR-4662b, hsa-miR-1284). Patients harbouring *KRAS* 3′UTR variants associated with altered miRNA binding profiles exhibited significantly shorter overall survival, suggesting a possible association between disrupted miRNA–*KRAS* interactions and adverse clinical outcomes [101].

Smoking modulates the expression of microRNAs. To investigate the relationship between oncogene mutations and miRNA expression profiles in lung cancer patients, tumour tissues were analysed. KRAS-mutated tumours were associated with 13 significantly upregulated miRNAs. Analysis with TargetScan identified hsa-miR-15b-3p and hsa-miR-21-3p as candidate miRNAs predicted to target *KRAS*. Furthermore, independent of the mutation status of *KRAS* and other oncogenes, four miRNAs (miR-187-5p, miR-147b, miR-2861, and miR-6075) were identified as promising survival markers for patients with lung cancer [102].

miR-21 requires particular caution because it is widely considered an oncogenic miRNA in NSCLC. In KRAS-driven lung-tumour models, miR-21 overexpression enhances tumourigenesis, whereas genetic deletion can partially suppress tumour formation, consistent with a predominantly pro-tumourigenic role mediated through repression of negative regulators of RAS/MEK/ERK signalling and apoptosis [103,104]. It should therefore not be presented as a simple KRAS-directed tumour suppressor.

The translational interpretation of KRAS-regulating miRNAs is limited by the predominance of cell-line and murine xenograft studies, supraphysiological miRNA overexpression, model-specific target repertoires, and incomplete information on pharmacokinetics and off-target effects. Therapeutic development will require tumour-selective delivery and durable intracellular exposure. Approaches under investigation include lipid or polymeric nanoparticles, engineered extracellular vesicles, viral vectors, and chemically modified oligonucleotide mimics or inhibitors, each with distinct trade-offs in stability, tissue specificity, immunogenicity, and toxicity [105,106].

A summary of the *KRAS*-associated miRNAs discussed above, including their reported roles and the principal limitations of the available evidence, is provided in Table 3.

#### 5.1.2. CircRNA and lncRNA

Compared with miRNAs, evidence for circRNAs and lncRNAs in KRAS-driven NSCLC is less mature but rapidly expanding. These molecules can regulate KRAS-associated pathways through miRNA sponging, transcript stability, transcriptional feedback loops, and extracellular-vesicle signalling. Their relative stability and tissue-specific expression also make them attractive biomarker candidates, although prospective clinical validation remains limited [107,108].

CircRNAs and lncRNAs also play diverse roles in tumourigenesis and may promote cancer development by sponging tumour-suppressive miRNAs, increasing oncogene transcription, and enhancing the stability of oncogenic transcripts [80].

Higher expression of circ_0000620 was observed in LUAD tissues and cell lines compared with adjacent normal tissues and normal human bronchial epithelial cells, respectively. circ_0000620 functioned as a molecular sponge for miR-216b-5p, whereas miR-216b-5p directly targeted *KRAS* by binding to its 3′UTR. Moreover, METTL3 (Methyltransferase 3) regulated the stability and expression of circ_0000620 through m6A modification. This study demonstrated that the METTL3/circ_0000620/miR-216b-5p/KRAS axis modulates cisplatin sensitivity in LUAD cells. Li et al. suggest that this signalling axis may represent a potential therapeutic target for overcoming cisplatin resistance in LUAD [109].

Exosomal circPTK2 and circHIPK3 expression was elevated in metastatic NSCLC patients, suggesting their potential utility as blood-based biomarkers of advanced metastatic disease. Furthermore, circPTK2/circHIPK3 enrichment was associated with *KRAS*-related NSCLC progression, monocytic myeloid-derived suppressor cell subsets (Gr1^−^/CD11b^−^ and Gr1^−^/CD11b^+^) and M2 macrophage enrichment, indicating their involvement in immunosuppressive tumour microenvironment remodelling. These findings highlight a novel interaction between circRNAs and tumour-associated macrophages that may represent a potential therapeutic target in KRAS-related NSCLC [110].

Analysis of The Cancer Genome Atlas (TCGA) datasets, human tissue samples and cell lines showed that HIF1A-As2, a long non-coding RNA, was upregulated in KRAS-driven NSCLC and positively correlated with KRAS expression. KRAS was reported to promote HIF1A-As2 expression through MYC. Furthermore, HIF1A-As2, a KRAS-induced lncRNA, was found to form a double-regulatory loop with MYC that contributed to cell proliferation, EMT and tumour metastasis. Therefore, HIF1A-As2 was proposed as a potential therapeutic target in KRAS-dependent NSCLC [111].

Bioinformatic analysis of the lncRNA–miRNA–mRNA competing endogenous RNA (ceRNA) network in lung adenocarcinoma using TCGA data identified a ceRNA network comprising 24 lncRNAs, 21 miRNAs and 142 mRNAs. These mRNAs were significantly enriched in biological processes, cellular components and kinase-related molecular functions. Among the identified lncRNAs, several novel prognostic candidates were found in LUAD. LINC01600, LINC00662, and CASC15, were negatively correlated with progression-free survival, while MALAT1 was positively correlated. In addition, LINC01600, LINC00696, and CASC15, were associated with overall survival. Importantly, the expression of LINC01600 was connected with *KRAS* mutation, as well as metastatic status. These findings suggest that LINC01600 may act as an oncogenic lncRNA and participate in *KRAS*-related signalling pathways in LUAD [112].

Overall, current studies demonstrate that non-coding RNAs are important regulators of *KRAS* expression and *KRAS*-related signalling pathways in NSCLC. Through direct interactions with *KRAS* mRNA or indirect modulation of KRAS-associated regulatory networks, they may influence multiple processes involved in carcinogenesis, including proliferation, invasion, metastasis, and response to treatment. Although the clinical utility of ncRNA-based strategies requires further investigation, the available evidence suggests that these molecules may serve as promising biomarkers and potential therapeutic targets in *KRAS*-associated NSCLC.

### 5.2. Post-Translational Modification

KRAS undergoes several post-translational modifications (PTMs), including methylation, phosphorylation, ubiquitination, nitrosylation, and acetylation, among others. By regulating KRAS activity and localisation, these modifications represent potential targets for cancer therapy [18].

#### 5.2.1. Methylation and Ubiquitination

SETD7-mediated methylation promotes KRAS degradation. Chiang et al. demonstrated that SET domain-containing lysine methyltransferase 7 (SETD7) exhibits tumour-suppressive properties in *KRAS*-mutant NSCLC models and participates in post-translational regulation of KRAS. SETD7 methylated KRAS at lysine residues K182 and K184, which facilitated the recruitment of the E3 ubiquitin ligase RABGEF1 (RAB Guanine Nucleotide Exchange Factor 1), leading to KRAS ubiquitination and subsequent degradation. In *KRAS*-mutant NSCLC cell lines (H358 G12C and A549 G12S), SETD7 overexpression reduced cell proliferation and colony formation. Moreover, higher SETD7 expression was associated with improved prognosis in patients with LUAD. This study proposed that the combined expression of SETD7 and RABGEF1 may have prognostic value in *KRAS*-driven NSCLC [113].

Mass spectrometry analysis identified ubiquitination of KRAS at residues K104, K117, K128, K147 and R135, with K128 representing the most prevalent ubiquitination site. Combined in silico modelling with in vitro experiments demonstrated that ubiquitination at K128 creates an additional interaction interface for GAPs, including NF1 and RAS p21 Protein Activator 1 (RASA1). This modification enhanced GAP binding and promoted GAP-mediated GTP hydrolysis, thereby constraining RAS signalling. In LUSC, KRAS K128 ubiquitination was significantly reduced compared with normal tissue. Furthermore, LUSC tumours with low levels of K128 ubiquitination were enriched for senescence-associated gene signatures, suggesting a potential role of this modification in the regulation of senescence-related phenotypes. Using pancreatic cancer cells harbouring KRAS G12D, the study showed that loss of K128 ubiquitination enhanced tumour growth, indicating that K128 ubiquitination restrains KRAS-driven tumourigenesis. This effect was associated with suppression of the NF1-dependent RALGDS/RALB/TBK1 signalling pathway and negative regulation of the KRAS-driven autocrine cytokine circuit and senescence-associated secretory phenotype [114].

On the other hand, deubiquitination may increase KRAS stability and thereby contribute to NSCLC progression. Ubiquitin-specific peptidase 7 (USP7) was identified as a deubiquitinase that binds KRAS through its TRAF domain and removes K48-linked polyubiquitin chains from lysine 147 (K147). This modification increased KRAS protein stability and enhanced downstream RAS signalling. USP7 was shown to promote the proliferation and survival of NSCLC cells, whereas its inhibition reduced KRAS protein levels and suppressed cell growth. Furthermore, elevated expression of either USP7 or KRAS was associated with poorer overall survival in patients with NSCLC. These findings suggest that the USP7–KRAS axis may contribute to NSCLC pathogenesis and could represent a potential therapeutic target [115].

#### 5.2.2. Phosphorylation

Another post-translational modification of KRAS is phosphorylation. In HEK293 human embryonic kidney cells, SRC proto-oncogene non-receptor tyrosine kinase (SRC) was shown to phosphorylate KRAS at Tyr32 and Tyr64, located within switch I and switch II, respectively. This modification induced conformational changes in both switch regions and impaired the normal progression of the GTPase cycle. Consequently, phosphorylated KRAS displayed reduced affinity for downstream effector proteins, including RAF [116].

#### 5.2.3. Oxidation and Nitrosylation

Oxidative modification has also been observed in the KRAS protein. Analysis of KRAS-dependent NSCLC cell lines demonstrated that treatment with ferrocene complex (C_16_H_20_FeClNO) increased intracellular reactive oxygen species (ROS), inhibited cancer cell growth and disrupted KRAS plasma membrane binding and signalling. This suggests mediation by oxidative modification of the KRAS His95 residue, which appears to play an important role in regulating KRAS membrane association and the growth of KRAS-dependent tumour cells [117].

Free radical oxidants such as nitric oxide (NO) can activate RAS proteins through cysteine 118 (C118) by generating a thiyl radical intermediate at this residue. This reaction promotes the release of GDP and its replacement by GTP, resulting in RAS activation. The final product of the reaction is the formation of a covalent bond between NO and the thiol group of C118, known as S-nitrosylation. It was found that oncogenic KRAS can be inhibited through oxidation at C118 by the non-radical oxidant H_2_O_2_, both in vitro and in vivo. To investigate this mechanism, C118S (with serine) and C118D (with aspartic acid) substitutions were introduced. The C118S substitution rendered mutant KRAS insensitive to redox regulation by NO and H_2_O_2_, whereas the C118D substitution inhibited mutant KRAS signalling by preventing NO-mediated activation and by mimicking permanent oxidation at C118 [118].

#### 5.2.4. Acetylation

Histone deacetylases (HDACs) are key enzymes involved in epigenetic regulation through the removal of acetyl groups from histone and non-histone proteins, thereby influencing gene expression and protein function. HDACs are classified into four groups: classes I, II and IV comprise Zn^2+^-dependent enzymes, whereas class III consists of NAD^+^-dependent enzymes known as sirtuins (SIRTs; silent information regulator proteins). SIRTs primarily function as deacetylases. Due to their ability to regulate the acetylation status of proteins involved in oncogenic signalling, including KRAS, HDAC inhibitors have emerged as promising therapeutic agents in cancer treatment [119].

Acetylation of mutant KRAS may improve the efficacy of cisplatin- or erlotinib-based therapy in NSCLC. A study performed in human NSCLC cell lines in vitro and in mouse models in vivo demonstrated that the expression of SIRT1, a NAD^+^-dependent deacetylase, was elevated at both the mRNA and protein levels in mutant KRAS tumours and cell lines. Furthermore, mutant KRAS upregulated SIRT1 expression through the RAF/MEK/c-MYC signalling pathway, whereas SIRT1 enhanced mutant KRAS activity, thereby forming a positive feedback loop. Shin et al. showed that SIRT1 deacetylated mutant KRAS at lysine 104 (K104), resulting in increased KRAS activity. In contrast, the acetyltransferase p300 acetylated the same residue, which reduced mutant KRAS activity. Moreover, treatment with the SIRT1 inhibitor EX527 in combination with cisplatin or erlotinib enhanced antitumour effects and reduced tumour colony formation in vitro and in vivo. Targeting PTM of mutant KRAS through SIRT1 inhibition and enhancement of p300-mediated acetylation has been proposed as a potential strategy to improve the therapeutic efficacy of cisplatin or erlotinib in KRAS-mutant NSCLC patients [120].

In contrast, SIRT2 has been proposed to function as a tumour suppressor in KRAS-dependent tumours. Using a KRAS G12D mouse model, loss of SIRT2 accelerated the progression of KRAS-driven lung adenocarcinoma. Mass spectrometry analyses identified lysine 147 (K147) as a reversible acetylation site specifically targeted by SIRT2. Acetylation of K147 enhanced KRAS activity and promoted cell proliferation, colony formation, cellular transformation and tumour growth [121].

Overall, post-translational modifications represent an important mechanism regulating KRAS function. The growing understanding of PTM-mediated control of KRAS activity and signalling may contribute to the development of novel therapeutic strategies for KRAS-driven cancers.

**Figure 6:**
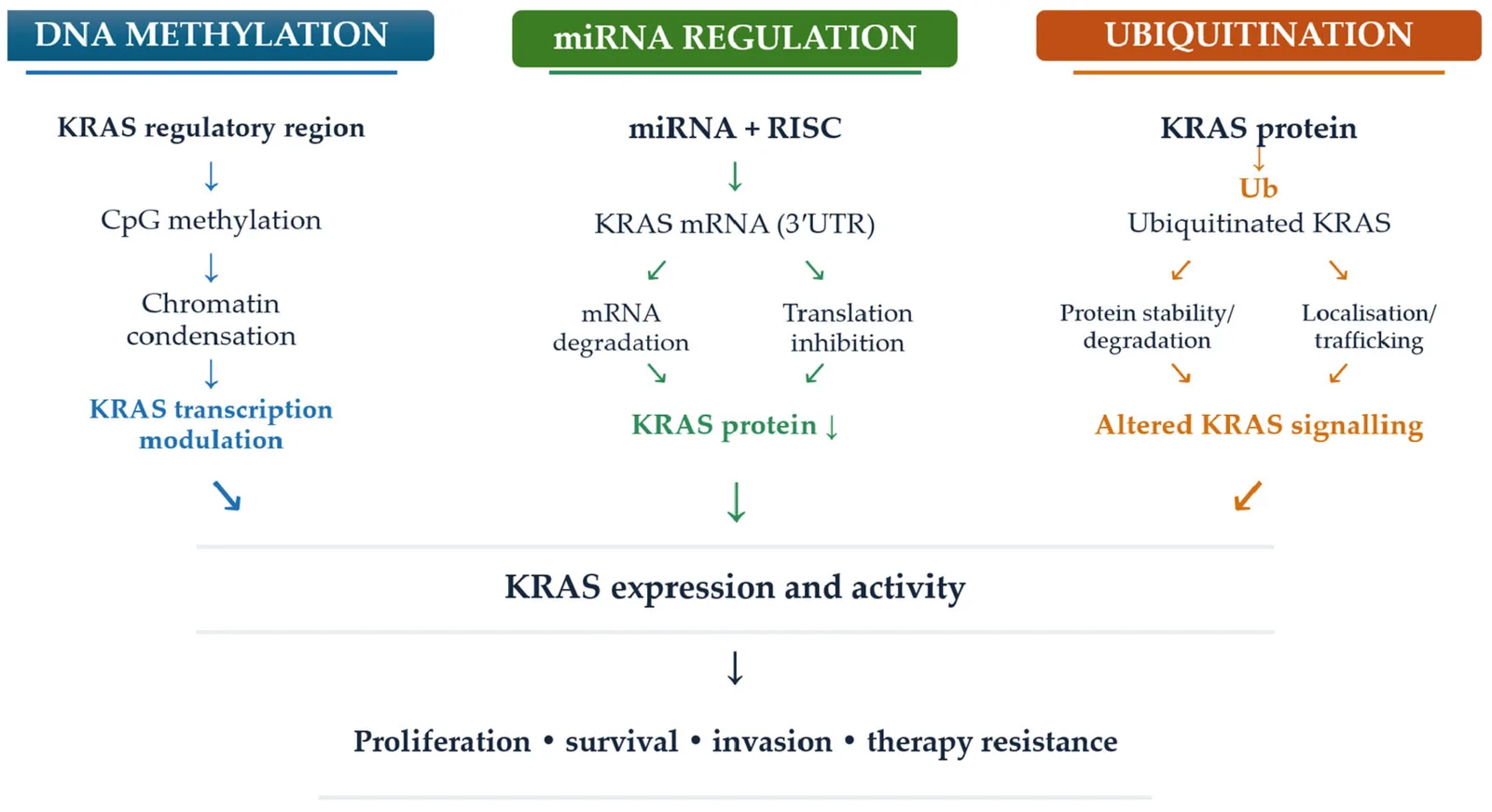
Multilevel regulation of KRAS expression and activity. DNA methylation may modulate KRAS transcription through alterations in chromatin organisation and accessibility. At the post-transcriptional level, miRNAs incorporated into the RNA-induced silencing complex (RISC) interact with the 3′ untranslated region (3′UTR) of KRAS mRNA, promoting mRNA degradation and/or translational inhibition. At the post-translational level, ubiquitination may regulate KRAS protein stability, degradation, localisation, trafficking, and signalling activity. Together, these mechanisms contribute to the regulation of KRAS expression and activity and may influence tumour cell proliferation, survival, invasion, and therapy resistance.

KRAS regulation extends beyond gene mutations and involves several additional regulatory layers. DNA methylation can alter chromatin accessibility and transcription, miRNA-mediated mechanisms regulate KRAS mRNA stability and translation, whereas ubiquitination modulates KRAS protein stability, trafficking, and signalling activity. A schematic overview of these mechanisms is presented in Figure 6.

## 6. KRAS-Targeted Therapies in NSCLC

For many years, KRAS was considered an “undruggable” therapeutic target because of its high affinity for GTP and the apparent absence of suitable binding pockets for small-molecule inhibitors. A major advance in the field was the identification of the Switch-II pocket (S-IIP), which enabled the development of selective *KRAS* G12C inhibitors, including sotorasib (AMG 510) and adagrasib (MRTX849). These agents represented the first clinically effective targeted therapies for patients with *KRAS* G12C-mutated NSCLC and significantly expanded available treatment options for this population [122,123]. A search of the ClinicalTrials.gov database performed on 21 June 2026 using the terms “KRAS” and “non-small cell lung cancer” identified approximately 230 registered clinical trials evaluating therapeutic strategies that target KRAS signalling either directly or indirectly [124].

### 6.1. KRAS Inhibitors Targeting the GDP-Bound State, GTP-Bound State, and Pan-KRAS

First-generation KRAS G12C inhibitors, including sotorasib and adagrasib, covalently bind the mutant cysteine in the Switch-II pocket and preferentially stabilise the inactive GDP-bound state [125,126,127]. In CodeBreaK 100, sotorasib achieved an objective response rate (ORR) of 37.1%, median PFS of 6.8 months, and OS of 12.5 months; longer follow-up confirmed durable benefit in a subset of patients [128,129]. In the randomised phase III CodeBreaK 200 trial, sotorasib improved PFS compared with docetaxel in previously treated KRAS G12C-mutated NSCLC, although no overall survival advantage was demonstrated [130]. In the KRYSTAL-1 NSCLC cohort, adagrasib achieved a confirmed ORR of 42.9%, median PFS of 6.5 months, and median OS of 12.6 months [131], with later analyses further defining the influence of co-mutations and transcriptional states on response [132]. Sotorasib and adagrasib received accelerated U.S. Food and Drug Administration (FDA) approvals for previously treated KRAS G12C-mutated NSCLC [122,127].

An alternative therapeutic strategy involves targeting the active GTP-bound form of KRAS. Elironrasib (RMC-6291), for example, functions as a tri-complex inhibitor that promotes the formation of a complex between cyclophilin A and active KRAS G12C. This approach enables direct targeting of the active state of KRAS and may overcome limitations associated with the rapid nucleotide cycling of the protein [133].

**Figure 7:**
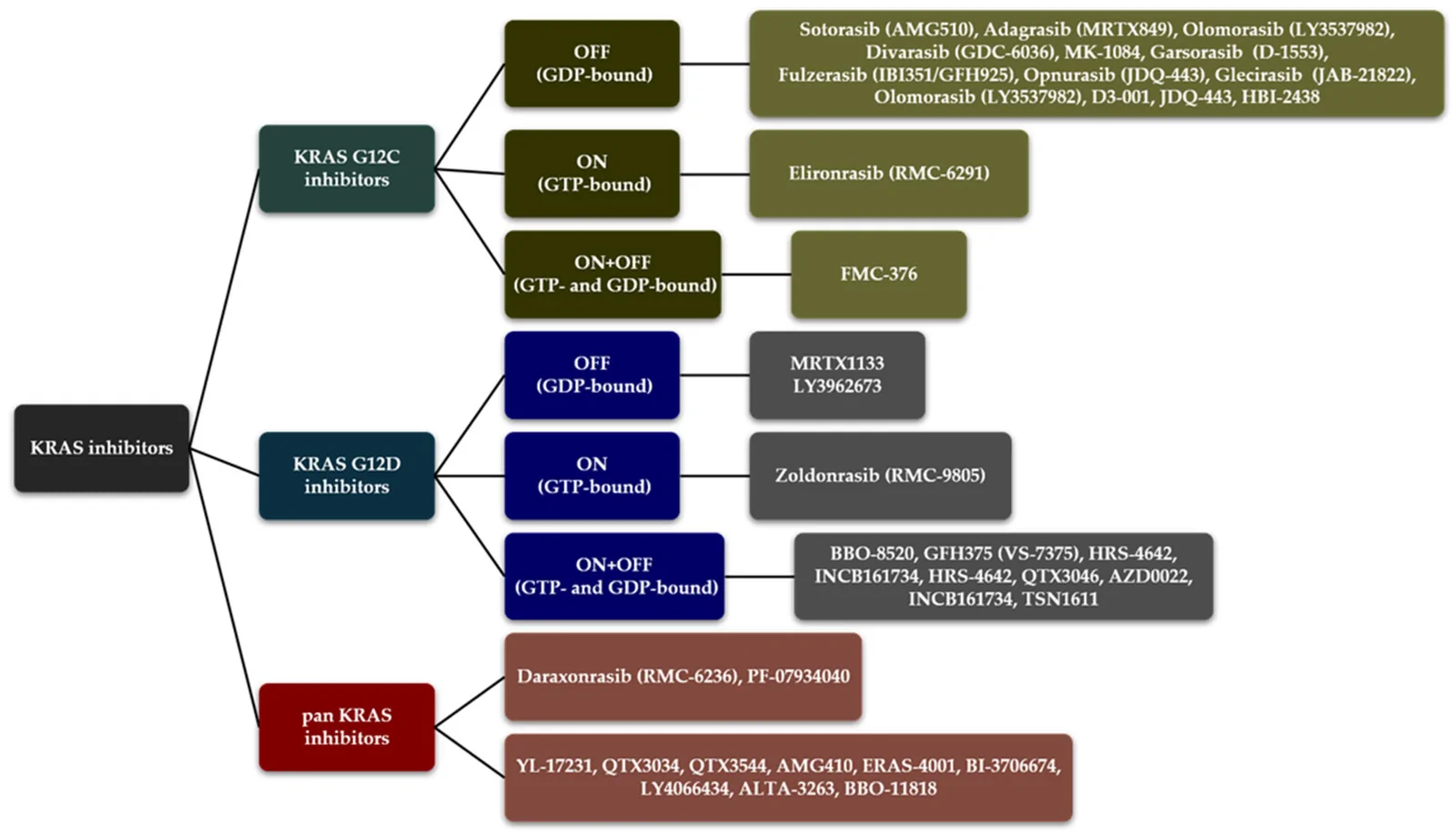
The major classes of KRAS inhibitors.

Multi-RAS inhibitors, also referred to as pan-RAS inhibitors, such as daraxonrasib (RMC-6236), are designed to target multiple *KRAS* variants, including G12D, G12V, and G12R, as well as wild-type RAS proteins. This strategy may extend targeted therapy to patients harbouring *KRAS* variants that are not susceptible to currently approved KRAS G12C inhibitors [134]. The major classes of KRAS inhibitors are illustrated in Figure 7 adapted from [127,135].

Daraxonrasib (RMC-6236) is an oral RAS(ON) multi-selective inhibitor that forms a tri-complex with cyclophilin A and active RAS, allowing inhibition across multiple RAS variants rather than a single codon substitution [134]. Clinical proof of concept has been reported in previously treated RAS-mutated pancreatic cancer [136], while ongoing clinical development also includes RAS-mutated NSCLC. For lung cancer, important translational questions include optimal sequencing, toxicity of broader RAS suppression, resistance mechanisms, and central nervous system exposure, because transporter-mediated efflux may limit brain penetration [137].

Therapeutic development is also expanding beyond G12C. MRTX1133 established preclinical proof of concept for potent, selective non-covalent KRAS G12D inhibition [138], while zoldonrasib (RMC-9805), an active-state covalent G12D inhibitor, has entered clinical evaluation [139]. For KRAS G12V, an EGFR-directed mutant-selective RNA-interference approach (EFTX-G12V) has demonstrated preclinical antitumour activity, including in lung cancer models [140]. These approaches remain investigational in NSCLC.

### 6.2. Combination Therapies

Although KRAS G12C inhibitor monotherapy has become the standard second-line treatment for patients with *KRAS* G12C-mutated NSCLC, the duration of clinical benefit remains limited. Consequently, increasing attention has been directed toward combination treatment strategies.

#### 6.2.1. Combination with Immunotherapy

*KRAS* mutations frequently co-occur with a high TMB, which has been associated with improved responsiveness to immune checkpoint inhibitors. Immunotherapy currently represents a standard first-line treatment option for many patients with KRAS-mutated NSCLC. Therefore, considerable efforts have been focused on evaluating combinations of KRAS inhibitors with antibodies targeting programmed cell death protein 1 (PD-1) or PD-L1 [141]. PD-L1 expressed on tumour cells suppresses antitumour immune responses through interaction with the PD-1 receptor on T lymphocytes. Preclinical and early clinical studies have demonstrated synergistic antitumour activity when sotorasib or adagrasib was combined with agents such as pembrolizumab or atezolizumab. These combinations have been associated with enhanced CD8+ T-cell infiltration within the tumour microenvironment [122,141]. Results from the KRYSTAL-7 study demonstrated an ORR of 63% among patients with high PD-L1 expression treated with adagrasib in combination with pembrolizumab [122].

#### 6.2.2. Combination with SHP2 and SOS1 Inhibitors

A major challenge in the treatment of *KRAS*-mutated NSCLC is the reactivation of RAS signalling through RTKs. Two proteins, SHP2 (Src Homology Region 2 Domain-Containing Phosphatase-2) and SOS1, play critical roles in this process. SHP2 participates in transmitting signals from activated RTKs to RAS, whereas SOS1 facilitates nucleotide exchange and KRAS activation. Consequently, inhibitors targeting SHP2 (e.g., TNO155 and RMC-4630) or SOS1 (e.g., BI-1701963) may prevent MAPK pathway reactivation and enhance the efficacy of KRAS inhibitors [123,142]. In a study evaluating the combination of the KRAS G12C inhibitor glecirasib (JAB-21822) with the SHP2 inhibitor JAB-3312, an ORR of 77.8% was observed, while disease control was achieved in more than 90% of patients. However, grade ≥3 adverse events occurred in approximately 42% of treated individuals [142,143].

#### 6.2.3. Combination with MEK Inhibitors

KRAS inhibition does not always achieve complete suppression of MAPK signalling. Therefore, vertical pathway inhibition, involving simultaneous targeting of both KRAS and MEK, has emerged as a promising therapeutic strategy. In the CodeBreaK 101 study, the combination of sotorasib and trametinib achieved disease control in 87% of patients [142].

#### 6.2.4. Combination with Chemotherapy

Various combination approaches are currently being investigated to overcome therapeutic resistance. The most common strategy involves the sequential administration of KRAS inhibitors following chemotherapy or standard chemoimmunotherapy. Alternatively, KRAS inhibitors may be administered concurrently with chemotherapy to achieve enhanced antitumour activity and greater tumour reduction. In the SCARLET study, the combination of sotorasib with carboplatin and pemetrexed achieved an ORR of 88.9% in the first-line setting, suggesting that KRAS inhibitors may also provide benefit when introduced earlier in the treatment course [144].

#### 6.2.5. Combination with EGFR-Directed Therapy

Adaptive receptor-tyrosine-kinase feedback, including EGFR signalling, can reactivate RAS-MAPK output after KRAS inhibition. This provides a mechanistic rationale for combining KRAS-directed agents with EGFR-targeted therapy. Such combinations are being actively investigated, but their role in NSCLC remains investigational and should be defined by prospective clinical trials [142,145].

#### 6.2.6. KRAS-Directed Immunological Approaches

Mutant KRAS can also be targeted as a shared neoantigen. Human leukocyte antigen-restricted T-cell receptor (TCR) approaches have demonstrated preclinical activity against KRAS G12V- and G12D-derived neoantigens [146,147]. In lung adenocarcinoma, a phase II study of the genotype-matched GI-4000 KRAS vaccine showed that the vaccine was well tolerated and induced mutant-KRAS-specific immune responses in 50% of evaluable patients, but the non-randomised study did not establish a definitive survival benefit [148]. KRAS-directed vaccines and TCR-based cellular therapies, therefore, remain investigational and require further validation in NSCLC.

### 6.3. Mechanisms of Resistance to KRAS-Targeted Therapies

Despite promising initial clinical responses, the durability of the benefit from KRAS G12C inhibitors remains limited, with median PFS typically ranging from 5 to 7 months. The principal causes of treatment failure include primary, acquired, and adaptive resistance mechanisms. These encompass alterations directly affecting KRAS (on-target resistance), activation of alternative signalling pathways (off-target resistance), tumour phenotypic plasticity, and epigenetic reprogramming. Understanding these mechanisms is essential for the development of more effective therapeutic strategies [149]. A recent synthesis of KRAS G12C resistance mechanisms and combination strategies in NSCLC is provided by Rosas et al. [145].

Primary resistance is defined as the absence of clinical benefit or very early disease progression following treatment initiation. This phenomenon is thought to result from limited dependence of tumour cells on KRAS signalling, as well as the presence of co-occurring molecular alterations, including mutations in *KEAP1*, *STK11/LKB1*, *SMARCA4*, and *CDKN2A* [150].

Acquired resistance develops following an initial response to therapy and is highly heterogeneous. On-target resistance mechanisms involve secondary alterations affecting the KRAS protein itself. Among the most extensively characterised are mutations involving the Switch-II pocket, including Y96C/D/H/N, H95D/G/N/R, and R68S. These alterations impair inhibitor binding and reduce the effectiveness of sotorasib and adagrasib. Additional *KRAS* mutations may also emerge during treatment, including G12A, G12D, G12R, G12S, G12V, G13D, Q61H, and Q61L. Because first-generation inhibitors selectively target KRAS G12C, they generally lack activity against these secondary variants [151,152]. Furthermore, amplification of the *KRAS* G12C allele has been observed in resistant tumours. Increased *KRAS* copy number results in higher levels of KRAS protein expression and a greater proportion of active GTP-bound KRAS, thereby reducing the ability of available inhibitors to achieve complete target suppression [153].

Off-target resistance results from reactivation of oncogenic signalling through KRAS-independent mechanisms. One of the most frequently observed processes involves activation of RTKs, including *MET* (mesenchymal–epithelial transition) amplification and activation of EGFR, HER2 (human epidermal growth factor receptor 2), and FGFR (fibroblast growth factor receptor 3) signalling. These alterations restore signalling through the RAF/MEK/ERK and PI3K/AKT/mTOR pathways despite effective KRAS inhibition. Mutations affecting genes involved in parallel signalling pathways have also been identified in patients following disease progression. These include alterations in *NRAS*, *HRAS*, *BRAF*, *MAP2K1* (mitogen-activated protein kinase kinase 1), *PIK3CA*, *PTEN* (phosphatase and tensin homolog), and *RET*. Such changes promote restoration of downstream proliferative signalling independently of KRAS G12C activity [154,155].

Emerging evidence suggests that resistance may also arise through non-genetic adaptive mechanisms. One prominent example is EMT, which enhances the migratory and invasive capabilities of tumour cells. EMT has additionally been associated with increased PI3K pathway activity, enabling tumour cells to survive despite KRAS inhibition [156].

Another adaptive mechanism involves histological transformation under therapeutic pressure. Lung adenocarcinoma may undergo transformation into squamous cell carcinoma or neuroendocrine tumour subtypes. Furthermore, transitions toward phenotypes resembling alveolar type I (AT1) cells have also been reported. These transitions are associated with profound alterations in tumour biology and may contribute to resistance against KRAS-targeted therapies [157,158].

## 7. Conclusions and Future Perspectives

KRAS remains one of the most challenging yet promising therapeutic targets in NSCLC. Growing knowledge of KRAS-dependent signalling networks, mutation-specific biology, epigenetic regulation, and post-translational modifications has substantially improved our understanding of tumour development and therapeutic vulnerabilities. The emergence of selective KRAS inhibitors has transformed the treatment landscape. However, intrinsic and acquired resistance continues to limit their long-term efficacy. Combination therapies targeting complementary molecular pathways and the tumour microenvironment represent a promising strategy to enhance treatment durability and patient outcomes. Continued investigation into the complex regulatory mechanisms governing KRAS activity, together with the identification of predictive biomarkers, will be essential for optimising personalised therapeutic approaches and expanding the clinical benefits of KRAS-targeted therapies.

In our view, the next phase of KRAS-directed treatment in NSCLC will require a shift from single-agent, single-mutation therapy toward dynamic molecular stratification. Priorities include improving the durability and central nervous system activity of KRAS G12C inhibition, clinically validating G12D-, G12V-, and multi-selective RAS-directed approaches, selecting combinations according to co-mutations and adaptive signalling, integrating ctDNA for longitudinal molecular monitoring [11], and determining where KRAS-directed vaccines or TCR strategies can add clinically meaningful benefit [127,134,136,137,138,139,140,145,146,147,148].

## Figures and Tables

**Table 1:** The clinical and biological characteristics associated with co-mutations.

Co-Mutation	Clinical Significance	Prognosis	References
*KRAS/TP53*	Inflammatory (‘hot’) tumour phenotype, higher programmed death-ligand 1 (PD-L1) expression, increased T-cell infiltration, often better response to immune checkpoint inhibitors	Relatively favourable vs. other *KRAS* co-mutations	[56]
*KRAS/STK11 (LKB1)*	‘Cold’ immune phenotype, primary resistance to immunotherapy, worse overall survival and progression-free survival	Unfavourable	[57]
*KRAS/KEAP1*	Resistance to oxidative stress, nuclear factor erythroid 2-related factor 2 (NRF2) pathway activation, metabolic adaptation, immunotherapy resistance	Very unfavourable	[57]
*KRAS/STK11/KEAP1*	Highly aggressive biology, poor response to immunotherapy, rapid progression risk	Very unfavourable	[57]
*KRAS/SMARCA4*	Aggressive clinical behaviour, shorter overall survival	Unfavourable	[58]
*KRAS/CDKN2A/CDKN2B*	Loss of cell-cycle control, increased proliferation, aggressive phenotype	Unfavourable	[57]

**Table 2:** Summary of reported *KRAS* sequence variants and their origins, study contexts, and major limitations.

Variant	Origin/Context	Population/Method	Main Reported Finding	Evidence/Limitation
rs61764370 (LCS6)	Germline 3′UTR polymorphism	Caucasian and Iranian cohorts; genotyping/meta-analysis	Conflicting associations with NSCLC risk; no consistent survival association	Population-dependent; inconsistent [73,74,75,76]
rs712	Germline 3′UTR polymorphism	Chinese COPD and Iranian cohorts	Reported association with lung-cancer susceptibility in selected cohorts	Needs replication across ancestries [77,78]
rs1137282	Reported germline variant	Resected NSCLC cohort	AA genotype associated with higher 3-year RFS	Single-cohort prognostic evidence [72]
rs121913240/Q61 variants	Frequently tumour-associated somatic variants	Tumour datasets/in silico analyses	Predicted functional impact; Q61 is an established oncogenic hotspot	Should not be labelled a germline SNP without matched normal DNA [68,69,70]
rs121913529/ G12D, G12V	Somatic oncogenic hotspot variants	Tumour sequencing datasets	Pathogenic KRAS driver substitutions	Somatic mutation terminology preferred [70,71]

**Table 3:** Summary of KRAS-related miRNAs discussed in this review, including their relationship to KRAS, experimental context, reported effects, and major limitations of the available evidence.

miRNA	Relationship to *KRAS*	Model/Evidence	Reported Effect	Key Limitation
let-7 family	Direct *KRAS* 3′UTR targeting	NSCLC tissues, cell lines, animal models	Reduced *KRAS* expression and tumour growth	Delivery and pleiotropic targets [85,93,94,95,96,97,98,99]
miR-1205	Direct *KRAS* targeting	A549/H1975; xenograft	Tumour-suppressive; reduced *KRAS*	Preclinical evidence [86]
miR-193a-3p	Direct *KRAS* 3′UTR targeting	Cell lines; in vivo models	Reduced proliferation/migration	Preclinical evidence [87,88]
miR-199b	KRAS feedback; multiple PI3K/ERK targets	NSCLC specimens and models	Suppresses *KRAS*-related signalling	Complex multi-target mechanism [89]
miR-181a-5p	Direct *KRAS* regulation	Murine/human tissues and cell lines	Reduced proliferation/migration	Limited clinical validation [90]
miR-202	Direct *KRAS* targeting	*KRAS*-mutant cell lines	Enhanced cisplatin-induced apoptosis	Preclinical evidence [91]
miR-101-3p	circ-MEMO1/miR-101-3p/KRAS axis	NSCLC models	Suppresses *KRAS*-driven progression	Indirect circRNA-dependent regulation [92]

## Data Availability

No new data were created or analyzed in this study. Data sharing is not applicable to this article.

## Abbreviations

The following abbreviations are used in this manuscript:

3′UTR	3′ untranslated region
AKT	Protein Kinase B
ALK	Anaplastic Lymphoma Kinase
BAD	BCL2-Associated Agonist of Cell Death
BRAF	B-Raf Proto-Oncogene, Serine/Threonine Kinase
CDKN2A/B	Cyclin-Dependent Kinase Inhibitor 2A and 2B
ceRNA	competing endogenous RNA
circRNA	circular RNA
COPD	Chronic Obstructive Pulmonary Disease
ctDNA	Circulating tumour DNA
DAG	Diacylglycerol
E2F1	E2F Transcription Factor 1
EGFR	Epidermal Growth Factor Receptor
ELK1	ETS Transcription Factor ELK1
EMT	Epithelial–Mesenchymal Transition
ERK	Extracellular Signal-Regulated Kinase
GAP	GTPase-Activating Protein
GDP	Guanosine Diphosphate
GEF	Guanine Nucleotide Exchange Factor
GRB2	Growth Factor Receptor-Bound Protein 2
GTP	Guanosine Triphosphate
HDACs	Histone deacetylases
HER2	Erb-B2 Receptor Tyrosine Kinase 2
HRAS	Harvey Rat Sarcoma Viral Oncogene Homolog
HVR	Hypervariable Region
IP3	Inositol 1,4,5-Trisphosphate
KEAP1	Kelch-Like ECH-Associated Protein 1
KRAS	Kirsten Rat Sarcoma Viral Oncogene Homolog
KSR2	Kinase Suppressor Of Ras 2
LCSs	Let-7 Complementary Sites
LKB1	Liver Kinase B1
lncRNAs	long non-coding RNAs
LUAD	Lung Adenocarcinoma
LUSC	Lung Squamous Cell Carcinoma
MAP2K1	Mitogen-Activated Protein Kinase Kinase 1
MDM4	Mouse Double Minute 4, Human Homolog Of; P53-Binding Protein
MET	MET Proto-Oncogene, Receptor Tyrosine Kinase
METTL3	Methyltransferase 3
miRNA	microRNA
mTOR	Mechanistic Target of Rapamycin
MYC	MYC Proto-Oncogene
ncRNA	Non-coding RNA
NF-κB	Nuclear Factor Kappa B
NF1	Neurofibromin 1
NGS	Next-Generation Sequencing
NO	Nitric Oxide
NRAS	Neuroblastoma RAS Viral Oncogene Homolog
NSCLC	Non-Small Cell Lung Cancer
ORR	Objective Response Rate
OS	Overall Survival
PCR	Polymerase Chain Reaction
PD-1	Programmed Cell Death Protein 1
PD-L1	Programmed Death-Ligand 1
PFS	Progression-Free Survival
PI3K	Phosphoinositide 3-Kinase
PIK3R1	Phosphoinositide-3-Kinase Regulatory Subunit 1
PIP2	Phosphatidylinositol 4,5-Bisphosphate
PIP3	Phosphatidylinositol-3,4,5-Trisphosphate
piRNA	PIWI-interacting RNA
PKC	Protein Kinase C
PLCƐ	Phospholipase C Epsilon
PLD	Phospholipase D
PTEN	Phosphatase and Tensin Homolog
PTM	Post-translational modifications
RABGEF1	RAB Guanine Nucleotide Exchange Factor 1
RAC1	Rac Family Small GTPase 1
RAF	Rapidly Accelerated Fibrosarcoma Kinase
RAL	Ras-Like GTPase
RALB	RAS-Like Proto-Oncogene B
RALGDS	Ral Guanine Nucleotide Dissociation Stimulator
RAS	Rat Sarcoma
RASA1	RAS p21 Protein Activator 1
RET	Ret Proto-Oncogene
RFS	Relapse-Free Survival
RHEB1	RHEB Pseudogene 1
RISC	RNA-Induced Silencing Complex
ROS	Reactive Oxygen Species
ROS1	ROS Proto-Oncogene 1, Receptor Tyrosine Kinase
RTKs	Receptor Tyrosine Kinases
SETD7	SET domain-containing lysine methyltransferase 7
SHP2	Src Homology Region 2 Domain-Containing Phosphatase-2
siRNA	small interfering RNA
SIRTs	Silent Information Regulator Proteins
SMARCA4	SWI/SNF-Related Matrix-Associated Actin-Dependent Regulator of Chromatin Subfamily A Member 4
SNPs	Single-nucleotide polymorphisms
SNVs	Single-Nucleotide Variants
SOS1	Son of Sevenless 1
SRC	SRC proto-oncogene non-receptor tyrosine kinase
STK11	Serine/Threonine Kinase 11
TBK1	TANK-Binding Kinase 1
TCGA	Analysis of The Cancer Genome Atlas
TCR	T-Cell Receptor
TIAM1	T-Cell Lymphoma Invasion and Metastasis 1
TMB	Tumour Mutational Burden
TP53	Tumour Protein p53
ULK1	Unc-51-Like Autophagy-Activating Kinase 1
USP7	Ubiquitin-Specific Peptidase 7
